# “We are not the same”: African women’s view of multipurpose prevention products in the TRIO clinical study

**DOI:** 10.2147/IJWH.S185712

**Published:** 2019-02-07

**Authors:** Mary Kate Shapley-Quinn, Kgahlisho N Manenzhe, Kawango Agot, Alexandra M Minnis, Ariane van der Straten

**Affiliations:** 1 Women’s Global Health Imperative (WGHI) RTI International, San Francisco, CA, USA, mshapley@rti.org; 2 Setshaba Research Centre, Soshanguve, South Africa; 3 Impact Research and Development Organization, Kisumu, Kenya; 4 School of Public Health, UC Berkeley, Berkeley, CA, USA; 5 Department of Medicine, Center for AIDS Prevention Studies, UCSF, San Francisco, CA, USA

**Keywords:** end-user research, HIV prevention, contraception, product preference, qualitative research

## Abstract

**Purpose:**

Unintended pregnancy and HIV infection present dual risks for young women in sub-Saharan Africa. New multipurpose prevention technologies (MPTs) are in development to simultaneously prevent unintended pregnancy and HIV, but there is a need for end-user research to ensure these products suit women’s needs. The Tablet, Rings and Injectables as Options (TRIO) for women study took place in Kisumu, Kenya, and Soshanguve, South Africa, with the goal of eliciting young women’s feedback on three potential MPTs.

**Methods:**

Women in TRIO used three placebo products that represented potential MPTs: daily oral tablets, monthly vaginal rings, and monthly dual injections in a randomized crossover design followed by a period in which they chose a product to use. Eighty-eight TRIO participants completed in-depth interviews and focus group discussions to understand their experiences using each product. Qualitative analyses were conducted after stratifying by product preference at the end of the crossover period.

**Results:**

The majority (65%) of participants preferred injections, with the remainder evenly split between tablets and rings. Discussions of preference for one product were closely linked with expressed dislike of another product’s attributes. Participants recognized heterogeneity in preferences and choices across users and stressed the need for multiple MPT options that confer a low burden on women’s daily lives.

**Conclusion:**

Rather than choosing a product to use based on the product’s perceived advantages, women’s choices were based on the unfavorable attributes of other TRIO products. Moreover, the importance that women placed on a given disadvantage varied. Disadvantages that women deemed as most important emerged as a greater driver of product preference than selecting products based on their advantages and favorable characteristics.

## Introduction

Unintended pregnancy and HIV infection constitute two challenges to achieving the United Nations 2030 Agenda for Sustainable Development in adolescent girls and young women. Despite the availability of a variety of contraceptive methods and growing options for HIV prevention, an estimated 26%–55% of pregnancies in sub-Saharan Africa are unintended,^1^ and in 2017, women aged 15–24 years accounted for a quarter of new HIV infections in this region.^2^ Novel multipurpose prevention technologies (MPTs) that combine HIV and unintended pregnancy prevention into a single delivery form would offer women a valuable tool – in addition to condoms – that could protect women from these dual risks.^3^

Multiple MPTs are being developed, including injectables, vaginal rings, gels, films, and inserts.^4^ Whereas developers of biomedical products are required to demonstrate safety and efficacy, experience in the field of reproductive health has also shown that end-users must be willing and able to use them for these technologies to achieve real-world effectiveness.^5^–^7^ Thus, it is essential to ensure that the voices of the intended users – in this case, women in sub-Saharan Africa – are heard and responded to in the development of these products. Hence, researchers in the MPT field have noted the need to incorporate end-user perspectives to assess the preference and inform the product development process, marketing, and roll out.^8^–^10^ Indeed, past research for HIV prevention and contraception has gained valuable insights when input from study participants was elicited.^11^–^13^

The Tablet, Rings and Injectables as Options (TRIO) for women study was designed to evaluate young women’s preferences among three placebo delivery forms (presented as potential MPT products) based on actual use.^14^ Placebo products were used as the main objectives of the TRIO study were to understand participants’ acceptability, preferences, and use of these three delivery forms, uncoupled from any possible side effects or their potential effectiveness. A qualitative component of TRIO was designed to further elicit women’s opinions of and experiences with these products. Here, we examine the barriers and facilitators that participants encountered in using each product. We describe the factors that participants considered when identifying the products that they did and did not prefer to understand why these factors differed among the TRIO participants.

## Methods

### Clinical study methods

The TRIO clinical study took place between December 2015 and December 2016 in Kisumu, Kenya, and Soshanguve, South Africa. Impact Research and Development Organization (IRDO) is based in Kisumu, the third largest city in Kenya, located along the shores of Lake Victoria in western Kenya. The Setshaba Research Centre (SRC) is situated in a residential area in Block H, Soshanguve Township in Tshwane South Africa. Participants in Kisumu and Soshanguve were recruited from urban or periurban communities near the IRDO and SRC using various strategies.

**Figure 1:**
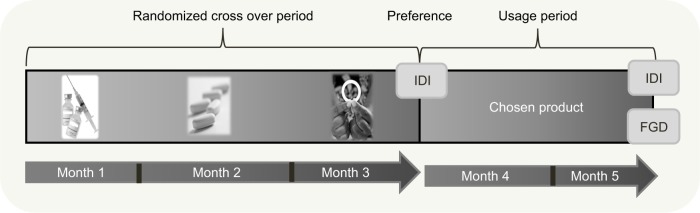
Timeline of product use and qualitative activities. **Abbreviations:** FGD, focus group discussion; IDI, in-depth interview.

In total, 277 women aged 18–30 years were enrolled in a two-staged, randomized, crossover study and asked to try three placebo forms of products that could potentially prevent pregnancy and HIV: a vaginal ring (inserted for 1 month), intramuscular injections (monthly dual gluteal injections), and daily oral tablets. After using each of these products for 1 month in a randomized sequence (stage 1: the crossover period), women were asked which product they would like to use again for the remaining 2 months of the study (stage 2: the usage period). No participants declined to use a TRIO product in the usage period. See Figure 1 for the study timeline. Participants in the TRIO study visited the study clinic monthly, during which time they completed interviewer-administered behavioral questionnaires, and were provided with condoms and risk-reduction counseling, and the clinicians completed clinical report forms. Further descriptions of eligibility criteria, recruitment, screening, engagement, and study procedures are described elsewhere.^14^,^15^

### Study products

As shown in Figure 1, the products provided to study participants were oral tablets (representing a co-formulated tablet similar to Truvada™; Gilead Sciences, Foster City, CA, USA) similar to those being rolled out worldwide for oral pre-exposure prophylaxis (PrEP);^16^ a vaginal silicone elastomer ring similar to the dapivirine ring used in recent clinical trials for HIV prevention (International Partnership for Microbicides, Silver Spring, MD, USA);^17^,^18^ and two 2 mL saline injections as used in the HPTN-076 trial.^19^ These were intended to closely resemble products commercially available or in the development pipeline for HIV prevention and MPT.

### Qualitative methods

The data coordinating center randomly selected a subset of 23 TRIO clinical study participants at each site to participate in a qualitative in-depth interview (IDI) at the end of the crossover period (at their month 3 visit) to collect information about experience with and preferences between the delivery forms, product attributes, and condoms. Participants were randomly selected to ensure that different product sequences during the crossover period were all represented in the IDIs. After the completion of stage 2 (the 2-month usage period), additional women were purposively recruited to further explore the attitudes about their chosen product, norms about MPTs, and perceptions of these delivery forms in their communities. Participants at each site were stratified by the product chosen at the start of the usage period. Participants who were exiting the study at similar times and who had chosen the same product were invited to a focus group discussion (FGD) to discuss how their experience with and preferences between products may have been the same or different – one FGD per product was conducted at each site (N=6). In addition, some participants were selected based on unique experiences, such as switching products during the usage period, to participate in a second round of IDIs (N=9). These IDIs were conducted to provide more information about use experiences and preferences that may not have been fully explored in the first round of IDIs.

The target sample sizes for these qualitative activities were chosen to provide adequate breadth and diversity of responses from among the clinical sample at each site, while ensuring that we reached data saturation (the point beyond which additional data collection would not yield new insights). In total, trained interviewers and facilitators conducted IDIs with 55 women (duration range: 30–112 minutes) and six FGDs with 37 women (duration range: 95–127 minutes).

Semi-structured guides were used for the IDIs and FGDs, which covered the following main topics: overall study experience, acceptability and use of study products, preferences between products, product choice, and recommendations for the future use of products and for product messaging (Table 1). Interviews and FGDs were audio recorded and conducted in English or a local language with which the participant(s) felt comfortable (Soshanguve: Tswana, Kisumu: Dholuo or Swahili). The audio recordings were transcribed and translated into English for coding and analyses.

The team developed a codebook that was based on codebooks used in prior studies^20^ and a conceptual model of HIV prevention product acceptability.^21^ A team of five analysts (four US-based and one South Africa-based) coded all transcripts using Dedoose, a web-based software for qualitative and mixed-method analysis. The average inter-rater reliability score (calculated as a pooled Cohen’s kappa) was 0.82. The coding team met regularly to discuss emerging themes, lack of agreement around code application, and needed code additions or modifications. After coding was completed, transcripts were stratified based on which product participants reported they most preferred on their behavioral survey completed at the month 3 visit, and code reports were generated for the following codes (or combinations of codes): “risk,” “barriers,” “facilitators,” “ring,” “injection,” “pill,” and “preference”. Given significant differences between the Kenya and South Africa sites in preference for the tablets and the injections,^14^,^22^ an additional analysis was carried out to examine any differences in these qualitative data by site.

### Ethical considerations

This study was conducted in accordance with the Declaration of Helsinki. All procedures and instruments were reviewed and approved by the KEMRI Scientific and Ethics Review Unit in Kenya and Pharma-Ethics in South Africa. All study participants provided written informed consent in a language that was understandable to them. The informed consent process detailed the purpose of using placebo products to understand delivery form preference and that participants’ aggregate responses would be shared with product developers to guide product development. Each participant was counseled that the study products provided no protection from HIV or pregnancy. All participants were encouraged to continue with their usual contraceptive method during study participation and counseled to use condoms (provided free of charge) for HIV and pregnancy prevention. Participants were informed of potential study-related risks such as discomfort with questions (and permission to decline to answer), study-related discrimination, loss of privacy, and risks related to use of the product forms (eg, discomfort swallowing tablets, pain or bruising at an injection site, or local irritation with ring use). Stringent procedures were followed to protect participant privacy, and staff were available to address any and all participant concerns with study-related product use and social risks. Participants received reimbursements for participation in qualitative activities worth KES 300/USD 3.00 (Kenya) and ZAR 100/USD 6.50 (South Africa).

## Results

Among the 88 women who participated in the qualitative component of the TRIO study, 45 were from Kisumu, Kenya, 43 from Soshanguve, South Africa, and the median age for the sample was 23 years. Four of these 88 participants completed both an IDI and an FGD. There were significant differences by country across many demographic and behavioral factors, including contraceptive method history, religiosity, education, and marital status (Table 2). We found no significant differences between the qualitative sample and the total clinical study sample in background characteristic examined (data not shown).

**Figure 2:**
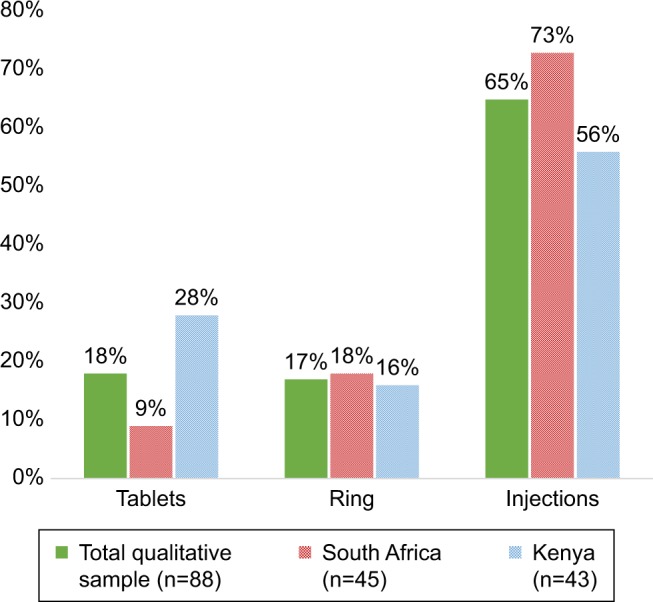
Month 3 product preference.

Product preferences among the qualitative sample were similar to those expressed by the TRIO clinical study sample overall:^14^ injections were most preferred at the month 3 visit (N=57); tablets and rings were preferred equally (N=16 and N=15, respectively) (Figure 2). Although the proportion of participants preferring the ring was similar across sites (eight participants in Soshanguve and seven in Kisumu), there were differences between sites for injections and tablets. Participants in Kisumu had relatively less preference for the injections than their counterparts in Soshanguve, and relatively greater preference for the tablets.

Interviewers and facilitators prompted participants to discuss the advantages and disadvantages of all three study products. However, the most salient themes that emerged were not tied to any one product or attribute. These crosscutting themes were the context of need for HIV and pregnancy prevention options, the desire for a variety of prevention options to meet the diverse needs of young women, a distinction between disadvantages of product use and barriers to product use, and an overarching preference for a product that would afford a low level of stress in their lives.

### Dual risks of HIV and unintended pregnancy

Overall, participants expressed an overwhelming preference for products with an MPT indication rather than just a contraceptive or HIV prevention indication. Participants at both sites spoke about perceptions around risk of HIV infection and unintended pregnancy in their communities and in their own lives, often describing situations that made them feel like they or their peers would be in need of an MPT product other than condoms. Many women in TRIO described difficulties with condom use that were intertwined with the dynamics of their relationships with male partners. Women also described interpersonal challenges with their male partners that they perceived as increasing their risk for unintended pregnancy and HIV. These included fears that use of family planning or condoms would be interpreted as indicating promiscuity, or suspicions around male partners’ truthfulness when talking about faithfulness or HIV status.

Participants in South Africa and Kenya spoke about the prevalence of HIV and unintended pregnancy as widespread problems in their broader communities as well. One participant in Soshanguve said, “…Teenage pregnancy is very serious in South Africa and most of the teenagers they don’t use – they don’t protect themselves, especially they don’t use condoms” (Injection preference, IDI), and a participant in Kisumu described her perception of rampant HIV in her community: “because I think almost ninety nine percent of the population is HIV positive” (Tablet preference, FGD). Participants at both sites also said they felt like, “…You’ll never know what will happen. Because women get raped out there” (Soshanguve, Injection preference, IDI) and would benefit from a product that would protect them from pregnancy and HIV.

### The desire for variety: “we are not the same”

Throughout the interviews and focus group discussions, participants dug into each of their personal experiences with the three TRIO products and how they felt about each one, but they also spontaneously raised the importance of offering a variety of prevention options. Women recognized that their personal preference would not necessarily mimic their peers’ preferences for an MPT product. One South African IDI participant stated, “We are not the same. Some they will like the ring. Some they will like the tablet … People are not the same. Some will talk. Some will like it. Some, they will never like it” (Injection preference), while another woman said in an FGD, “…As people we have different choices … I might not like the, the pills … and the injections but there’s other people who would” (Soshanguve, Injection preference). Similarly, in Kenya, two women in an FGD emphasized the importance of choice:

(#7) The ones that people will choose are different: I may like the ring that another may not like

(#6) …You just tell them there is a ring, a tablet and injections … [You will be given] the one that will be good to you…

In the analysis of participants’ experience with product use and how that impacted their preferences, it became apparent that discussions of preference for one TRIO product were deeply intertwined with the dislike of the alternate TRIO products’ attributes, and by association, similar products they may have used throughout their life (like oral contraceptives or medroxyprogesterone acetate injections). Particularly for the oral tablets and injections, women often described their rationale for preferring one of these products as a direct result of an undesirable experience with the other ones – whether it was an undesirable experience with the delivery form used in TRIO (tablets or injections), or a similar active product (eg, contraceptive tablets or injections) used previously.

The differences in participants’ descriptions of preference surrounding these two products – oral tablets and injections – were notable between the participants in Kisumu and Soshanguve. Specifically, differences by study site arose in the expression of strength of preference for the injections, the rationale behind a preference for injections over tablets, and descriptions of pain associated with administration of the injections. Choices were often described in relation to the lack of preference for another product, creating a narrative in which it was difficult to untangle whether the chosen product was truly desirable, or simply a default preference given that the other options were seen as less desirable. In this analysis, the attributes that were favored (eg, ease of use, comfort, discreetness) did not vary greatly between those who did and did not prefer a given delivery form, nor did they provide insights into reasons for preference, beyond low use burden and stress. In contrast, there were notable differences when analyzing the discussion of the disliked product attributes.

### Inconveniences or barriers to use? Divergent meaning of product disadvantages

Regardless of the product preferred, participants discussed common attributes that were viewed as advantages and disadvantages across all three TRIO products. For some participants, the disadvantages they noted for a given product were merely inconveniences, whereas for others, the same disadvantages were so salient that they became major barriers to using that product. Because unfavorable attributes of the TRIO products emerged as a greater driver of product preference than the favorable attributes, we focus the remainder of this section on the products’ disadvantages.

#### Tablets

When discussing TRIO tablets, many participants described two main disadvantages linked to a daily dosing regimen: privacy and adherence challenges. Participants who had not disclosed tablet use to others described how the presence of a tablet container created the opportunity for someone else to discover that they were taking medication. While using the tablets, one participant described her concern about a lack of privacy, saying:

…And maybe when you have somebody in the house and so you may not be able to … somebody may wonder what type of tablets you are taking … I had a visitor and I wondered how I will take the tablet and probably the house is just a single room… (Kisumu, Injection preference, IDI)

Other women expressed dismay with the daily dosing regimen saying that it limited their freedom by requiring they remember to take the tablet and be prepared to do so. This sentiment was more noticeable among participants in Soshanguve, who seemed particularly attuned to preserving their independence. In describing their rationale for preferring injections over tablets, separate participants in Soshanguve described wanting to be able to “do whatever I want to do,” “relax,” “just be myself,” and not wanting to carry a tablet container around with them.

Participants at both sites noted challenges with taking tablets daily: “…taking them around the same time is also a difficult thing to do. Maybe they could have just said that you just have to take them daily but any chosen time … But also the time – time strictness – now that time. It is so fixed!” (Kisumu, Injection preference, IDI). These challenges with the tablets were expressed across preference groups, but the perception of a constraint with fixed daily dosing regimen created a sense of stress and anxiety that was particularly pronounced among those participants who did not choose the tablets as their preferred product.

#### Ring

The ring was unique in that it was a novel product for participants, whereas most had previous experience with swallowing tablets or being injected. Participants described initial negative reactions when first seeing the ring. Importantly, for some participants, these concerns subsided over time with additional information about the ring and experience using it. As one participant in Soshanguve noted when asked what was scary about the ring:

The – uhm, the size of it. The thickness of it. Someway, somehow it – I had questions. Could it fit in there? … How will it feel during the actual [sex] act, you know. I had so many questions. But then after inserting it. Wow! I was surprised. (Ring preference, IDI)

Notably, participants who did not prefer the ring placed more emphasis on their concerns regarding ring use during menses [“I just felt it is not right to have it on while attending to my periods” (Kisumu, Pill preference, IDI)] and expressed heightened worries about having a foreign object in their body. Also salient among women who did not prefer the ring were discussions of possible negative experiences they anticipated, like the ring falling out unexpectedly, moving elsewhere in their body and getting lost, or inadvertent discovery of the ring by a male partner. Some of these ring concerns persisted despite overall positive ring use experience during the crossover stage, as was described by a participant who had to disclose ring use after her ring came out while having sex: “I like ring very much because once I have placed it I don’t have to worry again. But now the problem is, [laughs] at times there are men whose penis are too big that it sticks to the ring … It drops and now I have to explain. [Laughter]” (Kisumu, Ring preference, FGD).

#### Injections

When discussing concerns about injections, most participants raised the issues of the fear, pain, and side effects associated with injections. The minority of participants who did not prefer the injections were particularly attuned to these disadvantages, which were perceived as so salient that they became a barrier to use, rather than a mere inconvenience. One participant in Soshanguve described a pre-existing dislike of injections that persisted during the TRIO study, capturing the sentiment of those with an aversion to injections, and associated side effects and pain:

Respondent: …No, injections are not okay.

Interviewer: And then after getting injected, how did you feel about it?

R: Like I felt a little swelling after getting injected. After they had injected me with it.

I : Uhm, so it was painful?

R: Yes, it was painful, with the injection…

I: And after getting injected? Did you like it or you didn’t like it?

R: No, no. I have never liked injections; actually. I don’t like it. (Tablet preference, IDI)

#### Injections vs tablets: differing perspectives for South Africa and Kenya

Further differences between injection and tablet preferences were explored across sites due to the significant difference in preference by site. Three themes emerged: the perceptions around differing speeds of absorption between tablets and injections, a level of enthusiasm for the injections, and tolerance for the pain of the injections.

Participants in Soshanguve and Kisumu expressed interest in the injections because they saw them as offering the most privacy and placing a relatively low burden on the user. However, in Soshanguve, women were particularly interested in the injections due to their route of administration. For these participants, the oral route of administration for tablets was perceived as less efficient in achieving absorption and effectiveness, whereas they said the injection “…immediately gets in your body so it will circulate with blood tissues” (Soshanguve, Injection preference, IDI) and it “goes through the system and it moves faster … than the pills” (Soshanguve, Injection preference, IDI).

Overall, in Soshanguve women reported little ambivalence about the injections, frequently describing a strong preference for the injections due to their low user burden and convenience. However, among the participants in Kisumu, there was a more nuanced discussion around the benefits of the injections as a delivery method. One participant in Kisumu discussed a slight preference for the injections over the tablets but said that they were only “a little bit better” (Ring preference, IDI) than the tablets, and some others expressed near parity of preference for both the injections and the tablets. One participant described the injections as easiest to use because “It is done once … you need not repeat it” (Kisumu, Injection preference, IDI) yet still declared the tablets to be her most preferred product.

Although participants at both the sites expressed dismay with the pain they felt during the injections, participants in Kisumu were reportedly less tolerant of it. In both IDIs and FGDs at Kisumu, experience of acute pain and bleeding following the injection administration were discussed, and some participants even described their preference for the tablets as a result of their aversion to the pain of injections, despite feeling that the dosing regimen of the injections was preferable. One participant described the injections as the easiest product to use, but “The one I liked most of all the products was the tablet … Because it did not have any pain” (Kisumu, Tablet preference, IDI).

### The stress of a daily dosing regimen: a desire for longer-acting methods

As mentioned above and emergent across interviews, participants found that daily dosing regimens (like that of the TRIO tablet) caused stress or were “boring.” There was an overwhelming preference for products that were administered at least a month apart, but with a preference for an even longer duration. Participants described these longer gaps between dosing administration as allowing them to be “worry-free”: they wanted to avoid the anxiety of having forgotten a dose and desired a situation where it would be acceptable to forget about their prevention product for a long period of time. In discussing the times that she had accidentally skipped the tablets, one participant said, “…[Y]ou find that there are things that you cannot plan for like mine I never planned to go and sleep somewhere so you find that that day I missed my – one of the tablets that I was supposed to take. Yes. And carrying it in your bag wherever you want to go is very hard” (Kisumu, Ring preference, IDI).

A focus group participant in Kisumu used the example of a vegetable vendor to illustrate why a longer-acting product like the injection would be better than a daily tablet for some women:

…If you give tablets to the woman selling vegetables at the market, she will leave in the morning without taking the tablets, and she will come back late in the night so tired, she will just sleep … At least if she gets the injection, it will be good even if she forgets, she will just be okay … provided she remembers the return date for injection. (Tablet preference, FGD)

When discussing the injection – regardless of preference group – participants liked that the injection lasted throughout the month. Summarizing the benefits of a product that has a month-long duration, one participant in Kisumu who preferred the injection at her month 3 visit said, “…Once you are injected then you are done, you have nothing else to worry about” (FGD).

Participant comments highlighted an interest in prevention products that provide the security of continuous protection and that do not interfere with their daily life – without interruption of their normal daily activities and routine.

## Discussion

This qualitative study was embedded within a clinical study of the placebo forms of three potential MPT products in Soshanguve, South Africa, and Kisumu, Kenya. We sought to explore the barriers and facilitators that participants experienced while using the study products. Overall, participants expressed enthusiasm across the board for an MPT product. When participants discussed their own decision-making around product preference and choice in the TRIO study, the dislikes of study product attributes played a critical role in shaping product preferences – more so than the likes. In discussing the same disadvantages of a product, women who preferred that product found the disadvantages to be mere inconveniences, whereas other women considered the disadvantages to be so salient that they became barriers. These women would lean toward preferring a different product with disadvantages that they found less problematic. In other words, participants seemed to be driven away from a product due to the weight that person placed on the barriers to product use, rather than proactively choosing a product based on its perceived advantages.

Across the participants’ descriptions of each product, there were striking similarities between what women said were advantages and disadvantages, regardless of whether it was the product they had selected as most preferred or not. This suggests that, while women recognize the same attributes of products as being disadvantages and advantages, individual women weigh those factors differently, which may ultimately impact their preferences between products. As women indicated when they pointed out the intrinsic differences among them and their peers, there is no obvious choice that best suited all women.

The literature on women’s choice-making around contraceptive methods and a growing body of literature around choice in HIV prevention shows that a range of options to suit varied life circumstances or stages is critical for meeting the needs of the greatest number of women.^13^,^23^,^24^ As shown in the clinical and survey data in this same study,^14^,^22^ the majority of women did express a preference for injections in their stated preference, choice, and rating of the products. However, 35% of participants in this sample preferred the rings or tablets – not the injections – after trying each product. This paper further illuminates this range of preferences by describing the key factors that women considered when choosing which product they preferred.

We found that women made choices about the study products based on considerations such as privacy, burden in their day-to-day life, and physical sensations associated with product use, among others. Women did not place equal importance on these factors, and each product had characteristics that women saw as disadvantages. For women who considered those disadvantages to be important enough, these became barriers and it deterred them from choosing that product.

Specifically, for women who did not prefer the injections, they were often deterred by fear of needles, aversion to the pain associated with injections, and concerns about side effects. As an interesting addition to the discrete choice experiment and quantitative findings in TRIO, we found that women in Kisumu felt less enthusiasm for the injections than their counterparts in Soshanguve.^14^,^25^ The factors that contributed to women’s decisions not to choose the injections are important as there is little literature from the contraceptive field discussing the rationale for why women may not select injections when other contraceptive choices are available, as an option for prevention.

The concerns women voiced about the vaginal ring – fears around its size, use during menses, and worries about a foreign object in their body – are reflective of findings from other studies where women used vaginal rings. For example, women in the ASPIRE Phase III trial of a vaginal ring for HIV prevention also expressed early concerns about the ring due to its appearance and fears of what may happen while using it, although these eased over time,^20^ and in another study of a placebo ring, women also reported concerns about the ring coming out involuntarily or getting lost^26^ and difficulties in adhering to ring use were largely due to menses.^27^ The concerns women had with the vaginal ring largely diminished over time in all these studies where women were followed longitudinally,^20^,^26^,^28^ and after >12 months of use in ASPIRE, the ring was preferred to many other alternative product forms.^13^ TRIO participants were required to use the vaginal ring for only 1 month and would only have used it for longer (2 months) if it was selected during the usage period. Though participants’ mean ratings of the vaginal ring in TRIO increased significantly after 1 month of use (more so than the same rating for either of the other two products) as reported elsewhere,^22^ the month-long period of use may not be long enough to attenuate the initial concerns that many participants had about an unfamiliar product, and that were allayed in studies where women used a vaginal ring for 3 months or longer.

TRIO participants’ aversion to the tablets was largely due to the daily physical presence the product had in their lives – women disliked the burden and the issues this caused for privacy. In addition, instructions provided by the clinic staff to take the tablets at the same time every day were often interpreted as a strict requirement that caused additional stress, rather than the intended purpose as a tool to help tablet-takers establish a routine to avoid missed doses. Though the stress around the time of dosing could be allayed with improved communication by staff, the constraints of fixed time dosing came up in other oral PrEP studies.^29^ Furthermore, the issues around adhering to a daily dosing regimen and privacy are commonly cited in literature, particularly as it pertains to adolescent girls and young women in sub-Saharan Africa.^6^,^30^–^32^

As described in other studies of preferences for a HIV prevention product,^13^,^33^ a major consideration that emerged in TRIO as women described their preferences and choice among the three study products was the desire for a product that reduced the stress women feel about needing to remember to be adherent. Whether it was remembering a daily pill, changing a ring every month, or returning to a clinic periodically for injections, participants expressed concern about the possibility of forgetting, and the ensuing risk to their own health. Though women thought that the injection offered the best chance of reducing this worry, they recognized it had other disadvantages that would deter some women from choosing it, and many said that the monthly frequency of injections in this study was too frequent to be required to return to the clinic.

In this analysis, women’s descriptions of the advantages they found with each TRIO product did not display as much variety, nor were they as illuminating, as descriptions of what drove women away from alternative options. As future studies of product acceptability and product preference include different sets of product options (and include the experience of side effects and potential protection with active products), researchers must continue to explore the disadvantages that end-users encounter. Qualitative research will play a critical role in understanding the nuances of when disadvantages are merely that, and when they operate as barriers to product desirability or adherence. Combined with quantitative measures that assess the acceptability of known product disadvantages and willingness to use, researchers can build a better understanding of how to optimize product characteristics so that they are more congruent with users’ lifestyles.

Though rollout campaigns for new contraceptive and HIV prevention products often focus on the benefits that can be gained by using a product, these findings suggest that just as important – if not more – is a willingness to grapple with the downsides that each option may have. Particularly for providers and institutions who may offer new MPTs to women, it will be critical to understand that the impact of a perceived disadvantage associated with an MPT may have concrete implications for interest and uptake of a new product, as well as the available alternative choices. Similar to the best practices for counseling patients about contraceptive options,^34^ the results of this analysis indicate that providers will play an important role in helping each patient assess whether the disadvantages of an MPT will constitute a barrier to use for her, with the understanding that not all downsides will have equal salience across potential MPT users. This is an important consideration for the roll-out of new MPTs, especially considering that, most likely, three MPTs will not be simultaneously made available for women to choose among.

### Strengths and limitations

As reported in other publications of the TRIO study,^14^,^22^ there were significant differences by site in terms of product preferences and choices. Site difference was not a primary research question for the qualitative component, and we may have missed other site differences during this analysis. An additional methodological consideration is in the sequencing of the quantitative collection of preference data and the qualitative data collection. Procedurally, the quantitative preference data were collected prior to the qualitative interviews (on the same visit or up to 2 weeks prior to the qualitative IDIs). It is possible that participants’ discourse around product preference could have reflected a desire to justify the previous selections made in the quantitative survey, rather providing standalone responses regarding their rationale for their choice and preference selections.

The placebo products used in the TRIO study allowed for an in-depth analysis of participant experiences with three delivery forms, but the addition of the side effects experienced with active product use – and the benefits of pregnancy and HIV prevention – may impact preferences in such a way that we could not assess in this study. On the other hand, a particular strength of the TRIO qualitative component was the ability to conduct in-depth analyses of participants’ product assessments based on actual use of each of the three delivery forms.

## Conclusion

The development of new MPTs offers the promise of expanding options available for the prevention of HIV and unplanned pregnancy to young women. This paper provides further insights into what women saw as disadvantages associated with the three delivery forms they used, and how women factored those disadvantages into their decision of which product to choose and use again. Though many women expressed desire for longer-acting products, not all women preferred injections. As with the oral tablets and the vaginal rings, the injections also presented disadvantages that deterred some women from choosing them for the final 2 months of participation in the study. As new MPTs move along the development pipeline and approach roll-out, there must be strategies in place that deal with the disadvantages women may encounter while using a given product. These strategies should offer proactive solutions for barriers to product use – at the community, facility, and individual level – rather than merely focusing on the potential advantages that new MPT products will offer to women.

## Figures and Tables

**Table 1:** Sample topics and questions from qualitative guides

General topic	Example questions/prompts from the IDI guides (round 1 and 2) and FGD guides
Experience in the study	• Tell me about your experience being part of TRIO so far. • How would you describe your role in TRIO to a friend?
Use of study products	• What sorts of things about the area you live in helped or hindered your ability to use the study product(s) as directed? • How did the people who are close to you or who live with you influence your use of the study product(s)?
Acceptability of study products	• How would you describe your reaction when you saw the [tablets/ring/injections] for the first time and over the month you used them? • What did you like and dislike about using the [tablets/ring/injections]?
Preferences between products	• Assuming they were equally protective, if you could use one of the three study products (tablets, injections, ring) or condoms to prevent both pregnancy and HIV in the future, what method would you choose and why? • If you were not planning to get pregnant, would you prefer to use a product that protects against both HIV and pregnancy, or would you rather use two different products – one for HIV prevention, and another for pregnancy prevention?
Product choice	• Tell me about the product(s) you chose to use and why you chose it (them). • At your last visit you were given the option to switch products. How did you decide what to do?
Recommendations for the future	• Which one of the three study products (tablets, injections, or ring), if any, do you think your friends would be most likely to use in the future? Which one would you be most likely to use? • In the future when these products are available with medicine, it is likely that to be able to use the product women would need to get tested for HIV regularly, for example, every 3 months. How do you think women will feel about this requirement?
Recommendations for product messaging	• If these products from TRIO were proven effective and made available with active ingredients in them, how would you promote them to your sisters and friends? • Once the product is developed, what do you think would be the best way to inform women that this product is available?

**Abbreviations:** FGD, focus group discussion; IDI, in-depth interview.

**Table 2:** Selected demographics of the qualitative sub-sample

	Soshanguve, RSA	Kisumu, Kenya	Total

	N=45	N=43	N=88

	%	%	%

Age, mean (years)			
Median (IQR)	24 (21–26)	23 (21–26)	23 (21–26)
18–24	62	67	65
25–30	38	33	35
Currently have a primary partner	98	93	96
Married or cohabiting**	2	47	24
Currently have a casual sex partner	11	28	19
Ever exchanged sex for money/goods/services/place to stay**	4	23	14
Completed secondary school*	64	42	53
Earns an income**	18	47	32
Attend religious services each week**			
Sometimes/often	78	98	88
Never/no religion	22	2	13
Contraceptive methods ever used^a^			
Male condom	98	93	96
Injectable**	82	56	69
Implants	24	40	32
Pills	24	30	27
Female condom*	7	23	15
IUD	11	5	8
Other	2	5	3
None	0	2	1

**Notes:**

* *P*<0.05;

** *P*<0.01.

a Can select more than one.

**Abbreviations:** IQR, interquartile range; RSA, Republic of South Africa.

## References

[1] Sedgh G, Singh S, Hussain R (2014). Intended and unintended pregnancies worldwide in 2012 and recent trends. Stud Fam Plann.

[2] UNAIDS (2018). Miles To Go: Closing Gaps, Breaking Barriers, Righting Injustices.

[3] Thurman AR, Clark MR, Doncel GF (2011). Multipurpose prevention technologies: Biomedical tools to prevent HIV-1, HSV-2, and unintended pregnancies. Infect Dis Obstet Gynecol.

[4] AVAC (2016). Multipurpose Prevention Technolgies (MPTs): An Introductory Factsheet.

[5] Farrington EM, Bell DC, Dibacco AE (2016). Reasons people give for using (or not using) condoms. AIDS Behav.

[6] van der Straten A, Stadler J, Montgomery E (2014). Women’s experiences with oral and vaginal pre-exposure prophylaxis: the VOICE-C qualitative study in Johannesburg, South Africa. PLoS One.

[7] Van der Elst EM, Mbogua J, Operario D (2013). High acceptability of HIV pre-exposure prophylaxis but challenges in adherence and use: qualitative insights from a phase I trial of intermittent and daily PreP in at-risk populations in Kenya. AIDS Behav.

[8] Young Holt B, Romano J, Manning J (2014). Ensuring successful development and introduction of multipurpose prevention technologies through an innovative partnership approach. BJOG: Int J Obstet Gy.

[9] Brady M, Tolley E (2014). Aligning product development and user perspectives: social-behavioural dimensions of multipurpose prevention technologies. BJOG: Int J Obstet Gy.

[10] Brady M, Manning J (2013). Lessons from reproductive health to inform multipurpose prevention technologies: don’t reinvent the wheel. Antiviral Res.

[11] Marrazzo JM, Ramjee G, Richardson BA, VOICE Study Team (2015). Tenofovir-based preexposure prophylaxis for HIV infection among African women. N Engl J Med.

[12] Luecke EH, Cheng H, Woeber K (2016). Stated product formulation preferences for HIV pre-exposure prophylaxis among women in the VOICE-D (MTN-003D) study. J Int AIDS Soc.

[13] van der Straten A, Shapley-Quinn MK, Reddy K (2017). Favoring “Peace of Mind”: a qualitative study of African Women’s HIV prevention product formulation preferences from the MTN-020/ASPIRE trial. AIDS Patient Care and STDs.

[14] van der Straten A, Agot K, Ahmed K (2018). The tablets, ring, injections as options (trio) study: what young African women chose and used for future HIV and pregnancy prevention. J Int AIDS Soc.

[15] Weinrib R, Minnis A, Agot K (2018). End-users’ product preference across three multipurpose prevention technology delivery forms: baseline results from young Women in Kenya and South Africa. AIDS Behav.

[16] AVAC (2018). Global PrEP initiation Tracker.

[17] Nel A, Martins J, Bekker LG (2018). Safety of a silicone elastomer vaginal ring as potential microbicide delivery method in African women: a phase 1 randomized trial. PLoS One.

[18] Baeten JM, Palanee-Phillips T, Brown ER (2016). Use of a vaginal ring containing Dapivirine for HIV-1 prevention in women. N Engl J Med.

[19] Bekker LG, Li S, Tolley E HPTN 076: TMC278 La safe, tolerable and acceptable for HIV pre-exposure prophylaxis.

[20] Montgomery ET, van der Straten A, Chitukuta M, MTN-020/ASPIRE Study (2017). Acceptability and use of a dapivirine vaginal ring in a phase III trial. AIDS.

[21] Mensch BS, van der Straten A, Katzen LL (2012). Acceptability in microbicide and PrEP trials: current status and a reconceptualization. Curr Opin HIV AIDS.

[22] Minnis AM, Roberts ST, Agot K, TRIO Study Team (2018). Young women’s ratings of three placebo multipurpose prevention technologies for HIV and pregnancy prevention in a randomized, cross-over study in Kenya and South Africa. AIDS Behav.

[23] Delany-Moretlwe S, Mullick S, Eakle R, Rees H (2016). Planning for HIV preexposure prophylaxis introduction: lessons learned from contraception. Curr Opin HIV AIDS.

[24] Ross J, Hardee K, Mumford E, Eid S (2002). Contraceptive method choice in developing countries. International Family Planning Perspectives.

[25] Minnis AM, Browne EN, Boeri M (2019). Young women’s stated preferences for biomedical HIV prevention: results of a discrete choice experiment in Kenya and South Africa. J Int AIDS Soc.

[26] van der Straten A, Montgomery ET, Cheng H (2012). High acceptability of a vaginal ring intended as a microbicide delivery method for HIV prevention in African women. AIDS Behav.

[27] Montgomery ET, van der Straten A, Cheng H (2012). Vaginal ring adherence in sub-Saharan Africa: expulsion, removal, and perfect use. AIDS Behav.

[28] Nel A, Bekker LG, Bukusi E (2016). Safety, acceptability and adherence of Dapivirine vaginal ring in a microbicide clinical trial conducted in multiple countries in sub-Saharan Africa. PLoS One.

[29] van der Straten A, Montgomery ET, Musara P (2015). Disclosure of pharmacokinetic drug results to understand nonadherence. AIDS.

[30] Hosek S, Celum C, Wilson CM, Kapogiannis B, Delany-Moretlwe S, Bekker LG (2016). Preventing HIV among adolescents with oral PreP: observations and challenges in the United States and South Africa. J Int AIDS Soc.

[31] Mack N, Evens EM, Tolley EE (2014). The importance of choice in the rollout of ARV-based prevention to user groups in Kenya and South Africa: a qualitative study. J Int AIDS Soc.

[32] Bekker LG, Gill K, Wallace M (2015). Pre-exposure prophylaxis for South African adolescents: what evidence?. S Afr Med J.

[33] Krogstad EA, Atujuna M, Montgomery ET (2018). Perspectives of South African youth in the development of an implant for HIV prevention. J Int AIDS Soc.

[34] Dehlendorf C, Krajewski C, Borrero S (2014). Contraceptive counseling: best practices to ensure quality communication and enable effective contraceptive use. Clin Obstet Gynecol.

